# Vortioxetine Treatment Reverses Subchronic PCP Treatment-Induced Cognitive Impairments: A Potential Role for Serotonin Receptor-Mediated Regulation of GABA Neurotransmission

**DOI:** 10.3389/fphar.2018.00162

**Published:** 2018-03-06

**Authors:** Alan L. Pehrson, Christian S. Pedersen, Kirstine Sloth Tølbøl, Connie Sanchez

**Affiliations:** ^1^Department of Psychology, Montclair State University, Montclair, NJ, United States; ^2^H. Lundbeck A/S, Copenhagen, Denmark; ^3^Department of Clinical Medicine, Aarhus University, Aarhus, Denmark

**Keywords:** vortioxetine, subchronic PCP, attentional set-shifting test, novel object recognition, novel object placement, serotonin, GABA

## Abstract

Major depressive disorder (MDD) is associated with cognitive impairments that may contribute to poor functional outcomes. Clinical data suggests that the multimodal antidepressant vortioxetine attenuates some cognitive impairments in MDD patients, but the mechanistic basis for these improvements is unclear. One theory suggests that vortioxetine improves cognition by suppressing γ-amino butyric acid (GABA)ergic neurotransmission, thereby increasing glutamatergic activation. Vortioxetine’s effects on cognition, GABA and glutamate neurotransmission have been supported in separate experiments, but no empirical work has directly connected vortioxetine’s cognitive effects to those on GABA and glutamate neurotransmission. In this paper, we attempt to bridge this gap by evaluating vortioxetine’s effects in the subchronic PCP (subPCP) model, which induces impaired cognitive function and altered GABA and glutamate neurotransmission. We demonstrate that acute or subchronic vortioxetine treatment attenuated subPCP-induced deficits in attentional set shifting (AST) performance, and that the selective 5-HT_3_ receptor antagonist ondansetron or the 5-HT reuptake inhibitor escitalopram could mimic this effect. Furthermore, acute vortioxetine treatment reversed subPCP-induced object recognition (OR) deficits in rats, while subchronic vortioxetine reversed subPCP-induced Object Recognition and object placement impairments in mice. Finally, subPCP treatment reduced GABA_B_ receptor expression in a manner that was insensitive to vortioxetine treatment, and subchronic vortioxetine treatment alone, but not in combination with subPCP, significantly increased GABA’s affinity for the GABA_A_ receptor. These data suggest that vortioxetine reverses cognitive impairments in a model associated with altered GABA and glutamate neurotransmission, further supporting the hypothesis that vortioxetine’s GABAergic and glutamatergic effects are relevant for cognitive function.

## Introduction

Dysfunction in cognitive domains such as executive function and memory are a common feature of psychiatric illness, including major depressive disorder (MDD; McIntyre et al., 2013). Cognitive impairments in MDD patients may predict poor treatment response (Dunkin et al., 2000; Withall et al., 2009), and are associated with reduced functional outcomes (Jaeger et al., 2006). Additionally, the most commonly used antidepressants have a limited ability to improve cognitive symptoms. As a result, these cognitive impairments tend to remain prominent after mood symptoms have begun to recede (Herrera-Guzman et al., 2009, 2010). Thus, MDD-associated cognitive dysfunction is increasingly viewed as an important target for pharmacological remediation.

Vortioxetine is a multimodal antidepressant that acts as an agonist at 5-HT_1A_ receptors, a partial agonist at 5-HT_1B_ receptors, an antagonist at 5-HT_1D_, 5-HT_3_, and 5-HT_7_ receptors, and as an inhibitor at the serotonin (5-HT) reuptake protein (SERT; Sanchez et al., 2015). A recent meta-analysis of three randomized placebo controlled clinical trials in MDD patients demonstrated that vortioxetine induced significant improvements compared to placebo in the digit-symbol substitution test, a putative measure of processing speed and executive function, independently of its effects on mood (McIntyre et al., 2016). Additionally, *post hoc* analyses from several clinical trials support a role for vortioxetine in improving executive function (Harrison et al., 2016). Clinical studies have also demonstrated that vortioxetine significantly improves memory performance in the Ray Auditory Verbal Learning test (Katona et al., 2012; McIntyre et al., 2014). These results are supported by non-clinical data (i.e., data from studies in animals) suggesting that vortioxetine can improve aspects of executive function (Wallace et al., 2014) and memory (Mork et al., 2013; du Jardin et al., 2014; Jensen et al., 2014; Pehrson et al., 2016a). Thus, it appears that vortioxetine is capable of producing modest but significant improvements in some aspects of MDD-related cognitive dysfunction.

The glutamate neurotransmitter system is the primary excitatory drive in the mammalian central nervous system and its integrity is crucial for proper cognitive function. In support of this notion, experimental manipulations that reduce glutamate receptor activation, such as the α-amino-3-hydroxy-5-methyl-4-isoxazolepropionic acid (AMPA) receptor antagonist CNQX (Pierson et al., 2015), the *N*-methyl-D-aspartate (NMDA) receptor antagonist MK-801 (Stefani and Moghaddam, 2010), or the metabotropic glutamate 5 receptor (mGlu5) receptor antagonist MPEP (Christoffersen et al., 2008), have cognition impairing properties. Conversely, manipulations that enhance glutamatergic activity at postsynaptic targets, such as the AMPAkine aniracetam (Bartolini et al., 1996), the NMDA receptor glycine site agonist D-cycloserine (Flood et al., 1992), or the mGlu5 receptor agonist CDPPB (Fowler et al., 2013), tend to improve cognitive function, at least within an optimal dose window.

This laboratory has undertaken a significant effort in understanding the mechanism of action underlying vortioxetine’s effects on cognitive function, and toward this end several mechanistic hypotheses have been advanced, which we do not consider to be mutually exclusive. Among these are hypotheses suggesting that vortioxetine improves cognitive function via increases in acetylcholinergic neurotransmission (Mork et al., 2013), a histamine/orexin-dependent mechanism (Smagin et al., 2016), or an indirect, regionally selective increase in glutamate neurotransmission (Dale et al., 2016; Pehrson et al., 2016b). Non-clinical experiments from this laboratory have since suggested that acetylcholinergic neurotransmission is probably not a major cause of vortioxetine’s cognitive effects after chronic administration (Pehrson et al., 2016a), while the histamine/orexin hypothesis remains untested.

But there is accreting mechanistic evidence that vortioxetine can modulate γ-aminobutyric acid (GABA) and glutamate neurotransmission. Electrophysiological evidence demonstrates that vortioxetine suppresses inhibitory GABAergic neurotransmission via its potent antagonist effects at 5-HT_3_ receptors (Dale et al., 2014), and concomitantly increases pyramidal neuron firing under acute or subchronic dosing conditions (Riga et al., 2016, 2017). However, there is currently little behavioral evidence specifically supporting the notion that vortioxetine’s effects on these neurotransmitter systems are relevant for cognitive function.

The subchronic phencyclidine (PCP) administration model has a long history in non-clinical neuroscience, and has been used extensively as a putative model of schizophrenia. This theoretical conception of the subchronic PCP (subPCP) model is based on early observations that humans who abuse PCP over long periods can develop symptoms mimicking some aspects of schizophrenia, including positive symptoms like paranoia and auditory hallucinations, and most importantly for our purposes, persistent cognitive dysfunction featuring impaired executive function and memory (Jentsch and Roth, 1999). Importantly, rodent studies of subPCP-induced cognitive impairments commonly replicate dysfunction in these cognitive domains (for example, Redrobe et al., 2012).

Our interest in the subPCP treatment regimen as a model of cognitive dysfunction was driven by its complex mechanistic effects on GABA and glutamate neurotransmission, rather than its theoretical conception as a model for schizophrenia. Subchronic treatment with non-competitive NMDA receptor antagonists is associated with a variety of changes in GABAergic neurotransmission. For example, several research groups have observed reductions in parvalbumin (PV) immunoreactivity (IR) (Pratt et al., 2008; Redrobe et al., 2012), and Kv3.1 channel expression after subchronic PCP treatment (Pratt et al., 2008), suggesting altered function in fast-spiking GABAergic interneurons such as cortical basket and chandelier cells. Additionally, repeated NMDA receptor antagonism is associated with increases in binding (Kim et al., 2000) or K_D_ (Beninger et al., 2010) at the orthosteric GABA_A_ receptor site, as well as altered expression of genes encoding GABA_A_ receptor subunits (Kim et al., 2000). Similarly, subPCP-treated rodents have an increased threshold for inducing long-term potentiation and increased inhibitory postsynaptic currents (IPSCs) recorded from pyramidal neurons in the *Cornu Ammonis* area 1 (CA1) hippocampal subregion (Nomura et al., 2016), as well as reduced uptake of 2-deoxyglucose in the frontal cortex (Pratt et al., 2008).

Observations of reduced parvalbumin expression and increased IPSCs recorded from pyramidal neurons apparently contradict one another. But these competing observations may suggest that the subPCP model is associated with a complex dysregulation of GABAergic neurotransmission featuring both increases and decreases in inhibitory tone, perhaps originating from different interneuron populations. Moreover, a complex dysregulation such as this could reasonably be expected to induce cognitive dysfunction. If this concept is true, then vortioxetine’s ability to suppress aspects of GABAergic neurotransmission and concomitantly bolster glutamate neurotransmission may represent a logical mechanism through which a portion of subPCP-induced cognitive impairments may be attenuated.

Thus, the purpose of the current study is to evaluate vortioxetine’s effects in the subPCP model, which represents a biological model of cognitive impairment produced by dysregulation of GABA and glutamate neurotransmission. We hypothesized that subPCP treatment would cause impairments in executive function and memory that would be associated with changes in GABA receptor binding, and further that these changes would be reversed by acute or subchronic vortioxetine treatment.

## Materials and Methods

### Animals

A total of 212 adult rats were used in the experiments presented here. Of these, 128 male Long-Evans rats were used in the attentional set shifting task, 36 male Sprague-Dawley rats (Charles River Laboratories, Wilmington, MA, United States) were used in the object recognition (OR) task, and 48 male Long Evans rats (Charles River Laboratories, Wilmington, MA, United States) were used in autoradiography. Additionally, a total of 48 adult male C57BL/6NCrlBr mice (Charles River, Germany) were used in OR and object placement (OP) experiments. All animals were group housed in temperature- (20 ± 2°C) and humidity-controlled (30–70%) facilities on a 12-h light/dark cycle (lights on at 6 am). All rodent home environments consisted of plastic shoebox cages and featured sawdust bedding and appropriate environmental enrichments. Except where noted otherwise, all animals had *ad libitum* access to food and tap water. Specific information for animals used in each type of experiment is listed below. All experimental procedures were conducted in a manner that was consistent with local guidelines on ethical research in animal subjects and were approved by the relevant government authority or institutional animal care and use committee prior to the start of experiments.

#### Rat Attentional Set Shifting Task

Rats used in the attentional set shifting task were housed 2 per cage type III Macrolon cages. The rats were given access to food (Rats and Mice Growth and Reproduction Diet, Shanghai SLAC Laboratory Animal Co., LTD., Songjiang, Shanghai, China) and water *ad libitum* until the day before the first training session, where the diet was removed. During training and testing, each rat received approximately 11 g of diet per day, given after the training and/or test sessions. Rats assigned to the sub-chronic vortioxetine study, wherein vortioxetine was administered via diet, had food from the appropriate drug condition *ad libitum* (Purina 5001 rodent chow with or without vortioxetine mixed into it, Research Diets, Inc., New Brunswick, NJ, United States) until the day before the first training session. During training and testing, rats received approximately 11 g of appropriate diet per day.

#### Rat Object Recognition Task, and Autoradiography Experiments

Rats utilized in the novel OR task, as well as those used in autoradiography, PCR and Western blot experiments were housed three per cage in Rat IVC Green Line Sealsafe plus cages (Tecniplast USA, Philadelphia, PA, United States). Rats were housed for approximately 1 month before initiation of experimental procedures. Except for the first week, the rats were handled once every second day.

#### Mouse Object Recognition and Object Placement Tasks

For the mouse Object Placement Recognition and Novel Object Recognition tests, mice were housed two per Techniplast type III low cages (Scanbur A/S Denmark) with aspen bedding (Tapvei; Brogaarden, DK). For environmental enrichment, animals were given plastic igloos (Bio-Serv Mouse Igloo) and aspen bricks (Tapvej; Brogaarden, Denmark).

### Compounds and Treatments

#### Compounds

[1-(1-phenylcyclohexyl)-piperidine hydrochloride (PCP)], vortioxetine HBr and vortioxetine lactate were synthesized by H. Lundbeck A/S. Modafinil was purchased from Seqouia Research Products (United Kingdom). GABA, SR-95531 HBr, and baclofen were purchased from Tocris Bioscience (Minneapolis, MN, United States).

#### Radioligands

[^3^H] muscimol (36.9-38.3 Ci/mmol) was purchased from Perkin Elmer, Inc. (Waltham, MA, United States), while [^3^H] CGP54626 (30 Ci/mmol) was purchased from American Radiolabeled Chemicals, Inc. (St. Lousi, MO, United States).

#### Treatments

All doses listed below refer to the base, rather than the salt, of the compound in question.

Subchronic PCP treatment: The subPCP administration regimen used in rats was conducted as described in (Goetghebeur and Dias, 2009), which is a reliable method for inducing deficits in our hands. Rats in the subPCP experiments received injections of 5 mg/mL PCP or vehicle (saline) s.c. at a 1 mL/kg injection volume twice per day for 7 days. Subsequently, rats had a 7-day washout period prior to the beginning of experiments. The mouse subPCP regimen was conducted as described in Pedersen et al. (2014). Briefly, mice received vehicle or PCP (1.3 mg/mL concentration, 10 mL/kg injection volume) s.c once per day for 14 days followed by a 7-day washout prior to experimentation.

In acute vortioxetine administration experiments in rats, vortioxetine HBr was dissolved in 20% 2-hydroxypropyl-β-cyclodextrin (20%CD; parenteral grade, Roquette America, Inc., Keokuk, IA, United States) and injected 1 h s.c. at a volume of 1 mL/kg. For acute vortioxetine experiments in mice, vortioxetine lactate (used due to higher solubility) was dissolved in 10% CD and administered s.c. at a 10 mL/kg injection volume. Acute vortioxetine doses ranged from 1 to 10 mg/kg, and this dose range was chosen because it approximates the clinically relevant dose range of 50–90% occupancy at the 5-HT transporter (SERT; see Stenkrona et al., 2013; Leiser et al., 2014). For chronic vortioxetine administration experiments, Vortioxetine HBr was incorporated into Purina 5001 rodent chow (Research Diets, Inc., New Brunswick, NJ, United States) at a concentration of 0.18 g vortioxetine/kg of food, 0.6 g vortioxetine/kg of food, or 1.8 g vortioxetine/kg of food p.o. as described elsewhere (Wallace et al., 2014; Waller et al., 2017). Again, this dose range was chosen in order to approximate the clinically relevant range of SERT occupancy (Wallace et al., 2014; Waller et al., 2017).

Modafinil was included in the current study as a positive control compound in the attentional set shifting task. The compound and dose was chosen for this purpose based on previous evidence that this compound can reverse the effects of subPCP treatment in the attentional set shifting task (Goetghebeur and Dias, 2009). Modafinil for acute administration was suspended in 0.5% methyl cellulose (Sigma, Denmark) to a concentration of 32 mg/mL and administered 30 min p.o. at a volume of 2 ml/kg, for a final dose of 64 mg/kg. Due to the short half-life of modafinil, animals in this treatment group received a second administration of modafinil halfway through the attentional set-shifting task. All doses refer to the active base of each compound.

Escitalopram and ondansetron were included in the current study in order to assess the effects of 5-HT transporter inhibition, and 5-HT_3_ receptor antagonism in the attentional set shifting task, respectively. Escitalopram and ondansetron were dissolved in saline at a concentration of 0.5 and 1.6 mg/mL, respectively, and administered 1 hr s.c. at a 1 mL/kg of body weight injection volume. These conditions yielded final doses of 0.5 or 1.6 mg/kg, respectively. These doses were chosen on the basis of previously reported target occupancy data. 0.5 mg/kg escitalopram 1 hr s.c. is expected to produce approximately 90% SERT occupancy (Jensen et al., 2014), and is thus considered a clinically relevant dose of this compound for MDD (Meyer, 2007). 1.6 mg/kg ondansetron is expected to produce 60% or greater occupancy at the 5-HT_3_ receptor (du Jardin et al., 2014).

### Behavioral Assessments

#### Attentional Set-Shifting Task

Three separate attentional set-shifting studies were executed, one examining the acute effects of vortioxetine administration, a second examining the effects of sub-chronic vortioxetine administration, and a third examining the effects of acute escitalopram or ondansetron treatments. Each test used male Long-Evans rats (Charles River, United States) aged 5–6 weeks when arriving at the research facility.

The attentional set-shifting test (AST) methods used here were adapted from Goetghebeur and Dias (2009). The apparatus used for the AST test was a 44 cm × 64 cm × 30 cm black non-transparent Perspex test box, divided into 3 equal-sized areas (a starting area separated from a choice area subdivided in two). In the choice area, two 11 cm diameter terracotta pots were placed recessed into the box floor (with a 2 cm lip remaining above the floor) and separated by a divider. Odor (oils from The Body Shop^®^, United Kingdom) and media cues (not previously presented to the rats) were added to the pots.

Beginning on day 3 after the cessation of PCP administration, rats were subjected to a habituation period and trained to dig for a diet reward (half a Honey Loop cereal, Nestlé^®^) in terracotta pots filled with sawdust. On day 7 of PCP washout, rats were subjected to a single training session in which they were required to discriminate between two pots in order to find the reward. These discriminations were either based on differences in the digging media or the pot’s odor. On day 8 rats were presented with a series of increasingly difficult discriminations: a simple discrimination (SD), a compound discrimination (CD), two intra-dimensional shifts (ID1, ID2), an intra-dimensional shift reversal (ID2R), an extra-dimensional shift (ED) and an extra-dimensional shift reversal (EDR; see **Table 1**). Rats were required to meet a training criterion before moving on to a new discrimination type (for specifics, see Statistical Analysis, below).

**Table 1:** Attentional set shifting task methods.

Discrimination type	Media	Odor name (The Body Shop)
Training	Paper confetti	Madagascan vanilla flower
	Paper Worms	Atlas Mountain Rose
Simple discrimination (SD)	Plastic Beads	No Odor
	Vermiculite	No Odor
Compound discrimination (CD)	Plastic Beads	White Musk
	Vermiculite	Sandalwood Ginger
Intradimensional shift 1 (ID1)	Lava Balls	Cranberry
	Pills	Satsuma
Intradimensional shift 2/Intradimensional shift reversal (ID2/ID2R)	Plastic caps	Vanilla and Tonka Bean
	Paper clips	Lavender
Extradimensional shift/Extradimensional reversal (ED/EDR)	Wall Plugs	Exotic
	Catrine Pearl Litter	Amazonian Wild Lily


*Order of discriminations and example stimuli.*

The first trial of each discrimination stage was a discovery trial in which rats were allowed to dig in both pots, and to self-correct if digging in the wrong pot. Rats were only given one discovery trial. The discovery trial was included in the trials to criterion if the rat dug first in the correct pot, but not if the rat self-corrected an incorrect initial dig. If a rat showed no interest in the pots (no digging or sniffing in either of the pots) for more than 15 min, it was returned to the home cage to rest for approximately 30 min before resuming the test.

In the acute vortioxetine study, prior to the start of behavioral experiments on Day 8 of washout from PCP treatment, animals were randomly administered either 20% CD (Veh), vortioxetine at 1, 3, or 10 mg/kg 1 h s.c., or 64 mg/kg modafinil 30 min p.o. as described above (included as a positive control). Thus, the treatment groups in the acute vortioxetine study were as follows: Veh/Veh, PCP/Veh, PCP/1 mg/kg vortioxetine, PCP/3 mg/kg vortioxetine, PCP/10 mg/kg vortioxetine, or PCP/64 mg/kg modafinil.

For the sub-chronic vortioxetine administration study, rats had *ad libitum* access to either vehicle food (Purina 5001) or Purina 5001 food infused with either 0.22 g vortioxetine/kg of food weight, or 1.8 g vortioxetine/kg of food weight. Food dosing lasted from the first day of sub-chronic PCP administration and until the last day of the washout period (total of 14 days). After the end of the washout period where behavioral testing started, rats were fed with the same conditioned diet, however, under food restriction (∼11 g of diet per day per rat). In addition, some PCP-treated animals were fed vehicle food, but received acute injections of either 10 mg/kg vortioxetine, or 64 mg/kg modafinil, as described above. The treatment groups for the subchronic vortioxetine administration study were as follows: Veh/Veh, PCP/Veh, PCP/0.22 g/kg vortioxetine food, PCP/1.8 g/kg vortioxetine food, PCP/10 mg/kg vortioxetine 1 h s.c., or PCP/64 mg/kg 30 min p.o.

A third experiment was conducted to examine the effects of acute SERT inhibition or 5-HT_3_ receptor antagonism on subPCP-induced AST impairments. Acute dosing was conducted as described above for the acute vortioxetine dosing experiment, however, animals were randomly assigned to receive acute vehicle, escitalopram, ondansetron, or modafinil treatment. Thus, the treatment groups on this experiment were as follows: Veh/Veh, PCP/Veh, PCP/1.6 mg/kg ondansetron 1 h s.c., PCP/ 0.5 mg/kg escitalopram 1 h s.c., or PCP/64 mg/kg modafinil 30 min p.o.

#### Rat Object Recognition Task

The behavioral procedures for the rat OR task were conducted as described in du Jardin et al. (2014). The rat OR task was performed in a 49 cm × 49 cm × 37 cm plastic open field apparatus. The room housing the open field apparatus was kept under consistent, dim lighting, with an intensity of approximately 5 lux, and a radio played music softly for background noise. Objects used in this experiment included round, green, glass vases (diameter 5.7 cm, height 12 cm) and round, white, tin cookie jars (diameter 6.5 cm, height 8 cm). This object pairing was validated prior to use, in order to ensure no inherent bias existed toward either object in the pair. OR task sessions were recorded by a digital camera situated above the open field. Exploration time for each object was recorded with a stopwatch from the videos. Exploration was defined as directing the nose toward an object at a close distance or touching the object. Sitting on the object was not considered an exploratory behavior. An animal was excluded from the study if it failed to accumulate a total exploration time > 6 s, failed to explore both objects, knocked an object over, or jumped out of the arena.

Rats used in the OR task were subjected to the sub-chronic PCP regimen as described above. After the 7 days washout period, NOR testing was performed as previously described by (Redrobe et al., 2012). On day 7 after cessation of PCP treatment, each rat was given a habituation trial, where it was individually placed in the open field for 5 min of free exploration. Beginning on day 8 of PCP withdrawal, rats received acute injections of vehicle or 10 mg/kg vortioxetine s.c. 1 h prior to the training session. Thus, the treatment groups in this experiment were as follows: Veh/Veh, PCP/Veh, or PCP/10 mg/kg vortioxetine.

In the training session, each rat was re-habituated to the open field for 1 min. The rat was then placed in a holding cage while two identical objects were positioned in the open field for the OR task information trial (IT). Subsequently, the rat was positioned facing the wall and allowed to freely explore for 15 min. Upon completion, the rat was returned to its home cage and remained in the testing room during the retention period. The retention trial (RT) occurred 45 min after completing the IT. Two objects were again placed in the arena, one identical to the objects used during training and one novel. The placement of the novel object was pseudo-randomly assigned (left or right) and counter-balanced for each treatment group. The rat was positioned facing the wall and was allowed to freely explore for 3 min.

#### Mouse Object Placement and Object Recognition Tasks

Object placement and OR tasks in mice were executed in Perspex boxes measuring 40 × 40 cm with dark gray walls and a white floor. The tests were conducted during the light phase, yet in a subdued light (≈20 lux) and with the radio turned on in the background. Each trial was recorder by a camera mounted above the test arenas and later scored manually.

Objects in the mouse OP task consisted of small stork sculptures made of plaster, which were 11.5 cm in height, with a roughly rectangle-shaped base 7.5 cm in length and 4.8 cm in width. For the mouse OR task, objects consisted of (1) small cylindrical pepper mills (purchased empty, having never contained spice) made of clear plastic (10.5 cm in height, 4.5 cm diameter), and (2) small salt shakers made of light blue glass (again, purchased empty; 11.5 cm in height, 4.5 cm diameter). All objects were purchased at the dollar store.

One batch of mice was tested in both OP and OR. At day 1 of the washout period (first day post sub-chronic PCP administration), an s.c. bolus injection of vortioxetine 10 mg/kg was administered. Afterward, vortioxetine (0.6 g/kg Purina 5001 rodent chow) was administered p.o., starting at day 2 of the washout period and throughout the washout and testing period. On the day of OP testing, animals had received the s.c. bolus injection plus seven days of the vortioxetine-infused diet. On the day of OR testing, mice had received the s.c. bolus injection plus 13 (and 14) days of vortioxetine infused food.

The OP protocol was a modified version of the protocol described in Ennaceur and Meliani (1992). Mice were placed individually in the test arena for a 4-min acquisition trial. During the IT, two identical objects were situated in each of the arena’s front corners, 10 cm from the walls. Upon completion, the mouse was returned to its home cage and remained in the testing room during the period. In the RT, which occurred 1 h after the completion of the IT, the same to objects were situated in the arena, however, one of the objects were placed in one of the back corners (10 cm from the walls), opposite to the object placed in the front corner. The position of the objects was pseudo-randomly assigned (left or right) and counter-balanced for each treatment group.

During the acquisition trial and the test trial, cues were mounted at all four walls, to allow mice to orient themselves in the test arena. Cues were made in office PowerPoint, where white shapes were placed on a black background (cue 1: Two stars, cue 2: three vertical stripes, cue 3; one big arrow and cue 4: three smileys).

The OR testing was performed by giving the mice a 3 min IT with two identical objects. Mice were then returned to their home cages for 1 h, before being placed back in the test arena facing the wall for a 3 min RT. During the RT, one object was replaced with a novel object. Object pairs were validated prior to use to ensure no bias existed toward one of the objects in the pair. The position of the novel object was pseudo-randomly assigned (left or right) and counter-balanced for each treatment group. Mice that did not explore each object for a minimum of 2 s were excluded from further analysis.

### *Ex Vivo* Autoradiography

Rats used in *ex vivo* autoradiography (EVA) experiments were randomly assigned to one of four experimental groups characterized by either subchronic saline or PCP (as described above) along with either vehicle or vortioxetine-infused food (1.8 g vortioxetine/kg food weight, as described in the attentional set shifting task). On day 8 of PCP withdrawal, rats were anesthetized using CO_2_ and killed by decapitation. Brains were quickly and gently removed from the skull, flash frozen on powdered dry ice, and stored at -20°C until sectioning. Later, 12 μm coronal sections were collected from an area ranging from approximately +4.2 mm to +2.3 mm anterior to Bregma (Paxinos and Watson, 1998) using a cryostat (MicroM, Germany) and were thaw-mounted on glass microscope slides. This range of coronal slices was chosen in order to study the prelimbic and infralimbic subregions of the medial prefrontal cortex, given the importance of these regions in tests of executive function such as the attentional set shifting task (Ng et al., 2007). After the tissue mounted on the microscope slides had dried thoroughly (in order to reduce damage from freezing), slides were packed into slide boxes with desiccator pellets, and stored at -20°C for use in EVA saturation binding and competition experiments.

Each of the EVA procedures used in this study are variations on a similar theme. On the day of an experiment, slide-boxes were defrosted under a constant stream of air at room temperature until the boxes were dry and equilibrated with the environment in terms of temperature. This step was undertaken in order to ensure brain tissue was not damaged by frosting due to a sudden change in humidity. Prior to any binding experiments, slides were preincubated in the appropriate preincubation buffer and dried at room temperature. Subsequently, tissue was incubated in an assay buffer containing a radioligand appropriate for the receptor of interest, then rinsed in assay buffer, and dried overnight in a vacuum desiccator. Specific information on the contents of the preincubation buffers, assay buffers and other assay conditions are listed in **Table 2**. Finally, slides were opposed to a tritium-sensitive phosphor screen (Fuji imaging plate, GE Healthcare, Pittsburgh, PA, United States) for 5 days before being read in a phosphorimager (Typhoon FLA7000, GE Healthcare). Analysis methods are listed below under statistical analyses.

**Table 2:** *Ex vivo* autoradiography experiment methods.

Target receptor	GABA_A_ receptor		GABA_B_ receptor	
		
Assay	Saturation binding	Competition binding	Saturation binding	Competition binding
Preincubation Buffer	50 mM Tris Citrate	50 mM Tris Citrate	120 mM NaCl	120 mM NaCl
	(pH = 7.1)	(pH = 7.1)	6 mM Glucose	6 mM Glucose
	150 mM NaCl	150 mM NaCl	20 mM Tris HCl	20 mM Tris HCl
			4.7 mM KCl	4.7 mM KCl
			1.8 mM CaCl_2_ 2H_2_O	1.8 mM CaCl_2_ 2H_2_O
			1.2 mM KH_2_PO_4_	1.2 mM KH_2_PO_4_
			1.2 mM MgSO_4_	1.2 mM MgSO_4_
Preincubation time	30 min	30 min	15 min	15 min
Assay Buffer	50 mM Tris Citrate (pH = 7.1)	50 mM Tris Citrate (pH = 7.1)	Same as preincubation buffer	Same as preincubation buffer
Radioligand	[^3^H]muscimol	10 nM [^3^H]muscimol	[^3^H]CGP54626	3 nM [^3H^]CGP54626
Competitor/Non-specific binding agent	1 μM SR95531	GABA	10 μM baclofen	GABA
Incubation time	40 min	40 min	2 h	2 h
Rinse (in assay buffer)	3 × 5 min	3 × 5 min	2 × 15 min	2 × 15 min
Rinse temperature	4°C	4°C	RT	RT


Competition binding experiments were conducted in order to determine the Ki of GABA at each receptor target. In these experiments, the concentration of the relevant radioligand remained constant, but varying concentrations of GABA (ranging from 0.3 nM to 100 μM in half log steps) was included in the assay buffer.

In saturation binding experiments, the concentration of the relevant radioligand in the assay buffer was systematically varied in order to determine the K_D_ and B_max_ of the receptor in question. At each radioligand concentration, the non-specific binding was determined on a separate slide by including a high concentration of a non-radioactive competitor ligand in the assay buffer along with the radioligand. For GABA_A_ receptor saturation binding experiments, [^3^H]muscimol was examined in quarter log steps ranging from 0.56 to 300 nM. For GABA_B_ receptor saturation binding experiments, [^3^H]CGP54626 was examined in quarter log steps ranging from 0.17 to 100 nM.

### Statistical Analysis

#### General Data Processing and Statistical Analysis

All data in this study was analyzed using similar overall procedures. First, the Lilliefors test was used to examine whether data was distributed normally. In cases where the data was normally distributed, the data was examined for outliers using Peirce’s Criterion (Ross, 2003). Finally, data was analyzed for group differences using a suitable analysis of variance (ANOVA) model, followed by Tukey–Kramer *post hoc* tests where appropriate. For all statistical tests, statistical significance was set at *p* < 0.05. All statistical analyses were conducted using R (R Core Team, 2017), except where noted below.

#### Attentional Set-Shifting Task

The primary dependent measure for the attentional set shifting test was the number of trials required for an animal to reach the criterion of six consecutive correct trials. Set shifting data was analyzed using a two-way mixed repeated measures ANOVA, with discrimination type as a repeated measures factor and treatment as a between-subjects factor. This analysis structure is commonly used for similarly designed AST experiments (Redrobe et al., 2012; Maeda et al., 2014).

#### Object Recognition and Object Placement Tasks

The primary dependent measure for the OR and object placement tasks was a novel object or novel placement preference score, which were calculated from raw object investigation times using the following equation: Preference Score = IT_N_/(IT_N_ + IT_F_)^∗^100, where IT_N_ represents the investigation time for the novel object or placement, and IT_F_ represents the investigation for the familiar object or placement. Total exploration times from the information and test trials were analyzed for OP and OR experiments. Preference score and total exploration time data was analyzed for treatment group differences using a one-way between-subjects ANOVA. This analysis structure is similar to that used in previous studies with similar experimental designs (du Jardin et al., 2014; Jensen et al., 2014).

#### Autoradiography Data

There were three primary dependent measures for the autoradiography data, all of which were derived from intensity measurements obtained by analyzing phosphor screens using ImageQuant software (GE Healthcare Life Sciences, Pittsburgh, PA, United States).

For GABA competition binding studies, the primary dependent measure was pKi. Ki values for competition experiments were calculated separately for each animal, then aggregated to estimate sample averages and error. Ki values were calculated via non-linear regression, assuming a one-site competition binding, using GraphPad Prism version 6 for Windows (GraphPad software, La Jolla, CA, United States). pKi values were then calculated via the following formula: pKi = -Log_10_(Ki).

For saturation binding studies, the primary dependent measures were pKD and Bmax. First, a standard curve was generated by measuring optical intensity from a slide of tritium standards (American Radiolabeled Chemicals, St. Louis, MO, United States) included in the slide cassette during exposure. A linear regression was performed to determine the relationship between intensity and bound radioactivity (measured on the standards in units of μCi/g). Next, average intensity values from the non-specific bound condition for each concentration of radioligand was subtracted from intensity values from the total bound condition to produce a measure of specific binding for each radioligand concentration. These optical intensity values were transformed into bound radioactivity (expressed as nCi/mg) measurements by interpolation from the standard curve noted above. Finally, these values were further transformed by multiplying by the inverse of the relevant radioligand’s specific radioactivity (expressed as nCi/fmol). Thus, the final bound radioactivity data was expressed as fmol of radioligand/mg of tissue. K_D_ and Bmax values were calculated from these specific bound radioactivity data via non-linear regression, assuming a one-site saturation curve, using GraphPad version 6. p*K*_D_ values were calculated from *K*_D_ values as described above for pKi.

## Results

### Attentional Set-Shifting Task in Rats

#### Acute Vortioxetine Experiments

**FIGURE 1:**
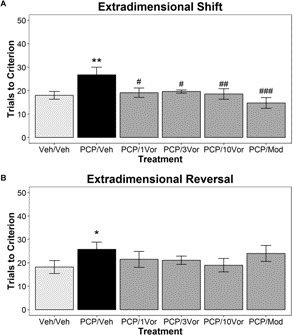
Acute vortioxetine administration reverses a subset of subchronic PCP (subPCP)-induced deficits in the attentional set shifting test (AST). SubPCP treatment induced significant impairments in the extradimensional shift **(A)** and extradimensional reversal **(B)** discriminations of the AST. Acute treatment with vortioxetine significantly reversed subPCP-induced impairments in the extradimensional shift at 1, 3, and 10 mg/kg 1 h s.c. Additionally, acute modafinil treatment at 64 mg/kg 30 min p.o. significantly reversed subPCP induced extradimensional shift impairments. However, neither acute vortioxetine treatment at these doses nor acute modafinil treatment significantly altered the subPCP-induced impairment in the extradimensional reversal discrimination. Data are presented as mean ± SEM. Asterisks represent significant differences from the Veh/Veh treatment group (^∗^*p* < 0.05; ^∗∗^*p* < 0.01). Number signs represent significant differences from the PCP/Veh treatment group (^#^*p* < 0.05; ^##^*p* < 0.01; ^###^*p* < 0.001). Final group sample sizes were as follows: Veh/Veh: *n* = 10; PCP/Veh: *n* = 12; PCP/1Vor: *n* = 10; PCP/3Vor: *n* = 10; PCP/10Vor: *n* = 10; PCP/Mod: *n* = 9.

**Figure 1** illustrates the effects of acute vortioxetine treatment on subPCP-induced impairments in the AST. The treatment main effect was non-significant [*F*(5,55) = 1.2, n.s.], however, we observed a significant main effect of discrimination type [*F*(6,330) = 92.95; *p* < 0.001], and a significant discrimination type × treatment interaction [*F*(30,330) = 2.079; *p* < 0.01]. *Post hoc* tests revealed a significant increase in trials to reach criteria in the ED discrimination stages in the subPCP/vehicle group compared to the vehicle/vehicle group (*p* < 0.01), indicating a subPCP-induced impairment. Within the ED discrimination (**Figure 1A**), this impairment was significantly reversed in the subPCP/acute vortioxetine treatment groups at the 1, 3, and 10 mg/kg 1 h s.c. doses, and in the subPCP/acute 64 mg/kg modafinil 30 min p.o. treatment group (*p* < 0.05 – 0.001). Within the EDR discrimination (**Figure 1B**), subPCP treatment induced significant increases in the number of trials required to meet criterion. However, neither acute vortioxetine nor modafinil significantly reversed subPCP-induced impairments in this discrimination.

#### Subchronic Vortioxetine Experiments

**FIGURE 2:**
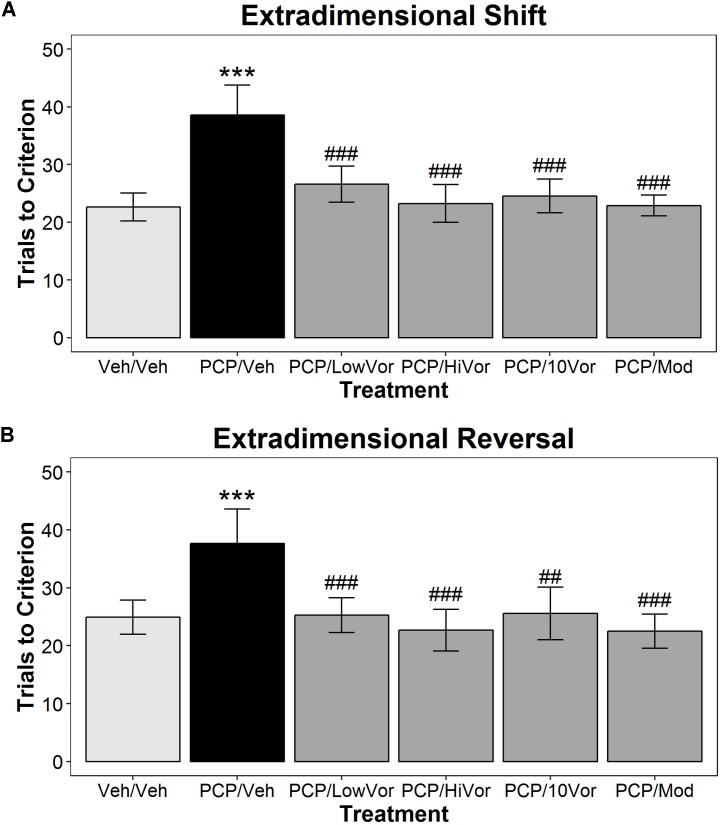
Subchronic vortioxetine treatment blocks subPCP-induced deficits in the AST. SubPCP treatment induced significant impairments in the AST extradimensional shift **(A)** and extradimensional reversal discriminations **(B)**. Subchronic treatment with vortioxetine at 0.18 g vortioxetine/kg of food weight (Low Vor), or 1.8 g/kg vortioxetine p.o. (Hi Vor) blocked subPCP-induced impairments in both discriminations. Additionally, acute treatment with 10 mg/kg vortioxetine 1 h s.c. or 64 mg/kg modafinil 30 min p.o. replicated its ability to reverse subPCP-induced extradimensional shift impairments, and also reversed subPCP-induced impairments in the extradimensional reversal test. Data are presented as mean ± SEM. Asterisks represent significant differences from the Veh/Veh treatment group (^∗∗∗^*p* < 0.001). Number signs represent significant differences from the PCP/Veh treatment group (^##^*p* < 0.01; ^###^*p* < 0.001). Final group sample sizes were as follows: Veh/Veh: *n* = 11; PCP/Veh: *n* = 12; PCP/LowVor: *n* = 12; PCP/HiVor: *n* = 12; PCP/10Vor: *n* = 9; PCP/Mod: *n* = 10.

**Figure 2** represents the effects of subchronic treatment with vortioxetine on subPCP induced impairments in the AST. For these data, we observed a significant main effect of treatment [*F*(5,60) = 3.08, *p* < 0.05], a significant main effect of discrimination type [*F*(6,360) = 119.64; *p* < 0.001] and a significant discrimination type × treatment interaction effect [*F*(30,360) = 2.1; *p* < 0.001]. *Post hoc* analysis revealed that subPCP treatment induced an impairment in AST performance, shown as a significant increase in trials to reach criteria in the ED (**Figure 2A**) and EDR (**Figure 2B**) discriminations (*p* < 0.001). In each case, the subPCP-induced impairments were significantly attenuated by treatment with subchronic low-dose (0.18 g vortioxetine/kg food weight) and high-dose (1.8 g vortioxetine/kg food weight) vortioxetine (*p* < 0.001). In addition, acute treatment with 10 mg/kg vortioxetine 1 h s.c. also induced a significant normalization of AST performance in subPCP-treated rats in both the ED and EDR tasks. Finally, acute treatment with 64 mg/kg modafinil significantly improved performance in both discriminations (*p* < 0.001 for all groups).

#### Acute Ondansetron and Escitalopram Experiments

**FIGURE 3:**
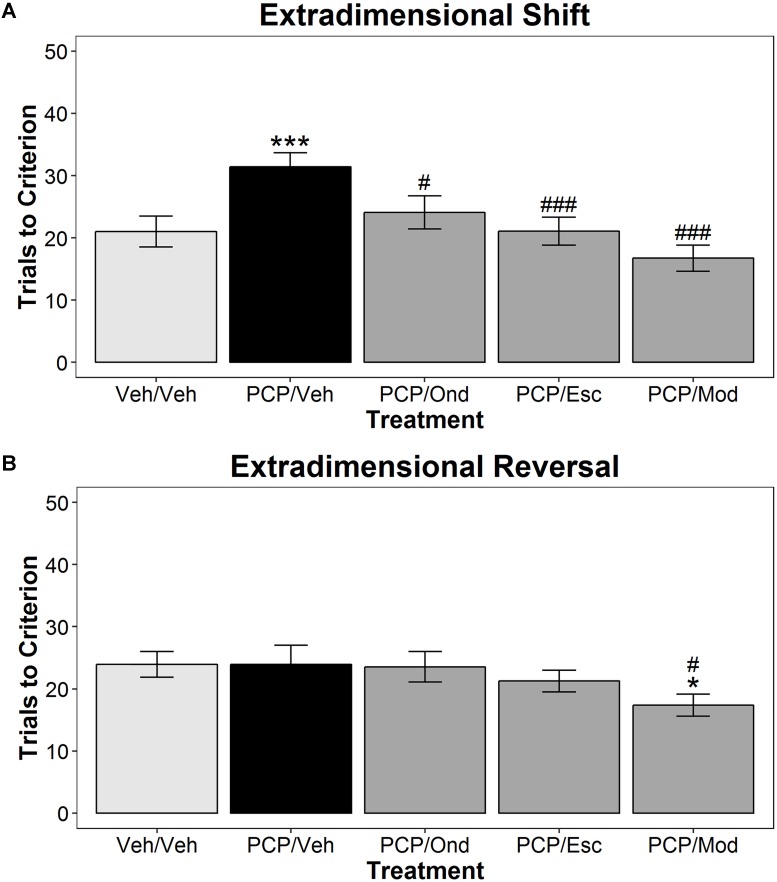
Acute treatment with ondansetron or escitalopram reverses subPCP induced deficits in the extradimensional shift discrimination of the AST. SubPCP treatment induced a significant impairment in the extradimensional shift discrimination **(A)**, but not in the extradimensional reversal **(B)**. Acute treatment with 1.6 mg/kg ondansetron (1 h s.c.) or 0.5 mg/kg escitalopram (1 h s.c.) significantly reversed the subPCP-induced deficit in the extradimensional shift. Acute treatment with modafinil also reversed subPCP-induced extradimensional shift impairment, and improved performance compared to both the Veh/Veh group and the PCP/Veh group in the extradimensional reversal discrimination. Data are presented as mean ± SEM. Asterisks represent significant differences from the Veh/Veh treatment group (^∗^*p* < 0.05; ^∗∗∗^*p* < 0.001). Number signs represent significant differences from the PCP/Veh group (^#^*p* < 0.05; ^###^*p* < 0.001). Final group sample sizes were as follows: Veh/Veh: *n* = 12; PCP/Veh: *n* = 12; PCP/Ond: *n* = 12; PCP/Esc: *n* = 12; PCP/Mod: *n* = 12.

**Figure 3** shows the effects of acute treatment with the 5-HT_3_ receptor antagonist ondansetron, the SERT inhibitor escitalopram, and modafinil on subPCP-induced impairments in AST performance. Once again, we observed a significant treatment main effect [*F*(4,55) = 2.97, *p* < 0.05], a significant discrimination type main effect [*F*(6,330) = 130.41, *p* < 0.001], and a significant discrimination type x treatment interaction [*F*(24,330) = 2.59, *p* < 0.001]. *Post hoc* testing in the ED discrimination (**Figure 3A**) found that subPCP induced a significant increase in the number of trials required to reach criterion compared to the vehicle/vehicle control group (*p* < 0.001). Additionally, subPCP-treated rats acutely administered ondansetron at 1.6 mg/kg (*p* < 0.05), escitalopram at 0.5 mg/kg (*p* < 0.001), or modafinil (*p* < 0.001) exhibited significant reductions in the trials to criterion dependent measure compared to PCP/vehicle-treated rats. In the EDR discrimination, subPCP treatment failed to induce a significant increase in the number of trials required to meet criterion compared to the vehicle/vehicle animals. Animals in the PCP/ondansetron or PCP/escitalopram treatment groups did not differ with respect to the trials to criterion dependent measure when compared with either the vehicle/vehicle or PCP/vehicle treatment groups. However, rats in the PCP/modafinil treatment group reached criterion in significantly fewer trials than animals in the vehicle/vehicle or PCP/vehicle groups (*p* < 0.05).

### Object Recognition Task in Rats

**FIGURE 4:**
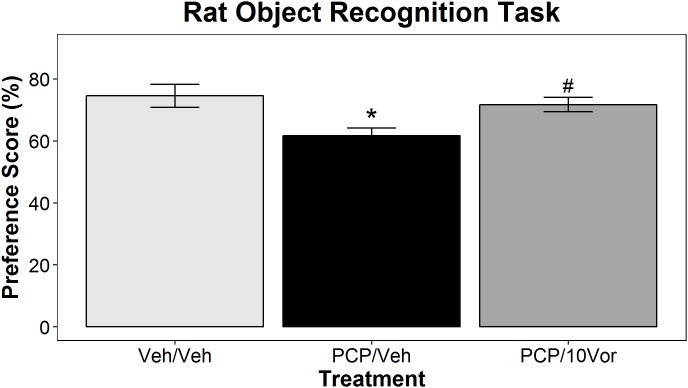
Acute vortioxetine reverses subPCP-induced object recognition task impairments. SubPCP treatment induced a significant deficit in object recognition performance, which was reversed by acute administration of 10 mg/kg vortioxetine (1 h s.c.). Data are presented as mean ± SEM. Asterisks represent significant differences from the Veh/Veh treatment group (^∗^*p* < 0.05). Number signs represent significant differences from the PCP/Veh group (#*p* < 0.05). Final group sample sizes were as follows: Veh/Veh: *n* = 11; PCP/Veh: *n* = 11; PCP/10Vor: *n* = 12.

In this portion of the study, we investigated the effects of treatment with subPCP alone or in combination with 10 mg/kg vortioxetine 1 h s.c. on OR performance in rats. Analysis of preference score data using one-way ANOVA revealed a significant main effect of treatment group [*F*(2,31) = 5.47, *p* < 0.01]. As shown in **Figure 4**, *post hoc* analysis revealed that subPCP treatment alone induced significant impairments in OR performance compared to vehicle-treated controls that was significantly attenuated by acute 10 mg/kg vortioxetine treatment (*p* < 0.05 for both effects).

Analysis of total exploration times in the training [*F*(2,31) = 0.7, n.s.] and test sessions [*F*(2,31) = 1.42, n.s.] revealed no significant group differences (see **Supplementary Table S1**).

### Object Recognition Task in Mice

**FIGURE 5:**
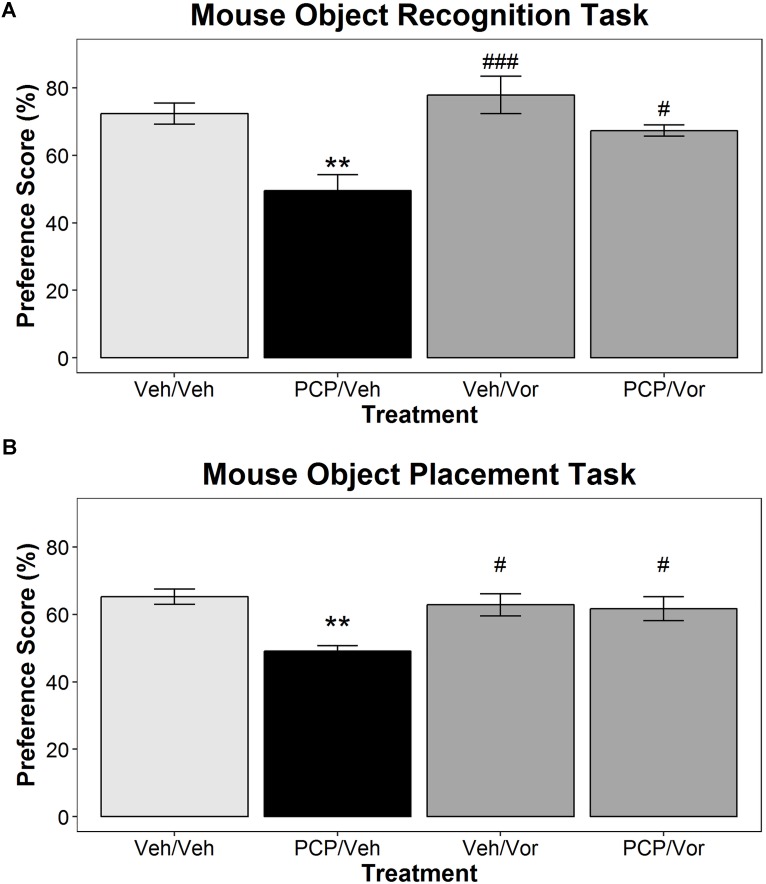
Subchronic vortioxetine treatment in mice blocks subPCP-induced impairments in the object recognition and object placement tasks. Subchronic PCP treatment induced a significant impairment in object recognition performance **(A)**, and object placement performance **(B)**. Animals in the vehicle/vortioxetine treatment group performed significantly better than subPCP/veh-treated animals in both tasks but did not perform differently from veh/veh controls. However, subPCP/vortioxetine-treated animals also performed significantly better than subPCP/veh-treated animals in both tasks. mean ± SEM. Asterisks represent significant differences from the Veh/Veh treatment group (^∗∗^*p* < 0.01). Number signs represent significant differences from the PCP/Veh group (^#^*p* < 0.05; ^###^*p* < 0.001). Final group sample sizes were as follows: object recognition: Veh/Veh: *n* = 11; PCP/Veh: *n* = 11; Veh/Vor: *n* = 11; PCP/Vor: *n* = 10. object placement: Veh/Veh: *n* = 11; PCP/Veh: *n* = 10; Veh/Vor: *n* = 12; PCP/Vor: *n* = 12.

**Figure 5A** represents OR task novel object preference scores in mice treated with vehicle/vehicle, subPCP/vehicle, vehicle/vortioxetine, or subPCP/vortioxetine. A one-way ANOVA revealed a significant relationship between treatment group and OR performance [*F*(3,40) = 9.19, *p* < 0.001]. *Post hoc* tests showed that animals in the subPCP/veh group had significantly lower novel object preference scores than control animals (*p* < 0.001). SubPCP/vortioxetine animals had significantly higher novel object preference scores than subPCP/vehicle animals (*p* < 0.01). There was no statistically significant difference in novel object preference scores between the vehicle/vehicle and vehicle/vortioxetine treatment groups.

Analysis of total exploration times in the training [*F*(3,39) = 0.6, n.s.] and test sessions [*F*(3,39) = 2.24, n.s.] revealed no significant treatment group differences (see **Supplementary Table S2**).

### Object Placement Task in Mice

**Figure 5B** shows OP task performance in mice in the same treatment groups noted above for the mouse OR task. Once again, a one-way ANOVA revealed a significant relationship between treatment group and object placement task performance [*F*(3,41) = 5.7, *p* < 0.01]. Further analysis demonstrated once again that animals in the subPCP/vehicle group had significantly reduced novel placement preference scores by comparison to vehicle/vehicle control animals (*p* < 0.01). Acute treatment with vortioxetine significantly attenuated the object placement task impairment induced by subPCP treatment (*p* < 0.05). Animals in the vortioxetine/vehicle group exhibited significantly higher object placement task performance than subPCP/vehicle-treated animals, but did not perform significantly better than vehicle/vehicle control subjects.

Analysis of total exploration times in the training [*F*(3,41) = 0.6, n.s.] and test sessions [*F*(3,41) = 0.59, n.s.] once again revealed no significant treatment group differences (see **Supplementary Table S2**).

### GABA_A_ and GABA_B_ Receptor Autoradiography in the Rat Medial Prefrontal Cortex

**Table 3** shows the effects of subPCP, vortioxetine or combinations thereof on pKi, pK_D_, and Bmax data for GABA_A_ or GABA_B_ receptor ligands in the prelimbic and infralimbic subregions of the rat medial prefrontal cortex.

**Table 3:** The effects of subchronic PCP, subchronic vortioxetine, or the combination on GABA receptor affinity and expression in the medial prefrontal cortex.

Treatment group	GABA_A_ receptors			GABA_B_ receptors		
		
	pK_i_	pK_D_	Bmax	pK_i_	pK_D_	Bmax
	(GABA)	([^3^H]muscimol)	(fmol/mg)	(GABA)	([^3^H]CGP54626)	(fmol/mg)
Veh/Veh (*n* = 12)	6.9 ± 0.05	7.7 ± 0.03	317 ± 10	6.4 ± 0.17	8.6 ± 0.04	541 ± 38
Veh/Vor (*n* = 11–12)	7.3 ± 0.04^a^	7.8 ± 0.06	302 ± 16	6.1 ± 0.14	8.6 ± 0.08	475 ± 34
PCP/Veh (*n* = 10–12)	7.1 ± 0.03	7.8 ± 0.03	296 ± 13	6.0 ± 0.10	8.7 ± 0.08	414 ± 33^a^
PCP/Vor (*n* = 11–12)	6.9 ± 0.08^c^	7.6 ± 0.03^a,b,c^	343 ± 18	6.2 ± 0.09	8.7 ± 0.06	342 ± 27^a,c^


*Subchronic treatment with vortioxetine increased the affinity of GABA for the GABA_*A*_ receptor, but when administered in combination with PCP, GABA’s affinity for this receptor was normalized. Additionally, subchronic treatment with the combination of PCP and vortioxetine induced a modest but significant reduction in the affinity of the [^*3*^H]muscimol for the GABA_*A*_ receptor. Neither PCP nor vortioxetine altered GABA_*A*_ receptor expression levels in this brain area (represented by Bmax). Neither PCP nor vortioxetine had any significant effects on the affinity of [^*3*^H]CGP54626 or GABA for the GABA_*B*_ receptor. However, treatment with PCP significantly reduced GABA_*B*_ receptor expression levels in this brain area, independently of vortioxetine administration. Values represent the average pK_*D*_, pK_*i*_, or Bmax for the relevant ligand ± SEM. ^*a*^Represents a statistically significant difference from Veh/Veh, ^*b*^Represents a statistically significant difference from PCP/Veh, ^*c*^Represents a statistically significant difference from Veh/Vor.*

#### GABA_A_ Receptors

Analysis of GABA pKi data at the GABA_A_ receptor uncovered a statistically significant main effect of treatment type [*F*(3,43) = 8.657, *p* < 0.001]. *Post hoc* analysis found that animals in the vehicle/vortioxetine treatment group had a significantly greater pKi compared to those in the saline/vehicle group (*p* < 0.01), representing an approximately 2.5-fold increase in the affinity of GABA for the GABA_A_ receptor. GABA pKi values from animals in the PCP/vortioxetine group were significantly lower than the vehicle/vortioxetine group (*p* < 0.01), but were not significantly different than other groups. SubPCP treatment on its own did not significantly alter GABA pKi values as assessed here.

Analysis of [^3^H]muscimol pK_D_ data also found a statistically significant main effect of treatment [*F*(3,42) = 7.849, *p* < 0.001], which was driven by a small but significant reduction in [^3^H]muscimol affinity for the GABA_A_ receptor in the PCP/vortioxetine group by comparison to all other treatment groups. No other significant group differences were observed in the pK_D_ data. Finally, analysis of Bmax values for [^3^H]muscimol found no significant differences [*F*(3,44) = 2.14, n.s.].

#### GABA_B_ Receptors

Analysis of GABA pKi and [^3^H]CGP54626 pK_D_ data for GABA_B_ receptors found no significant effects of treatment group [*F*(3,42) = 1.491, n.s. and *F*(3,42) = 1.982, n.s., respectively].

However a significant main effect of treatment was found in the [^3^H]CGP54626 Bmax data [*F*(3,42) = 6.4, *p* < 0.01]. Further analysis revealed that subPCP treatment led to significant reductions in Bmax values compared to the saline/vehicle group (*p* < 0.05). This reduction was maintained in PCP/vortioxetine treated animals (*p* < 0.001), however, no significant differences were observed between the PCP/vehicle and PCP/vortioxetine groups.

## Discussion

In the present study, we demonstrated that acute or subchronic vortioxetine treatment attenuated subPCP-induced impairments in the attentional set shifting task in rats, and that acute administration of either the 5-HT_3_ receptor antagonist ondansetron or the selective SERT inhibitor escitalopram were independently sufficient to recapitulate this effect. In addition, we demonstrated that acute vortioxetine reversed subPCP-induced impairments in the rat OR task, and that subchronic vortioxetine blocked subPCP-induced impairments in the mouse OR and placement tasks. Autoradiographic data suggests that subchronic vortioxetine treatment induced a modest but significant increase in the affinity of GABA for the GABA_A_ receptor that was not present when vortioxetine was co-administered with PCP. The combination of subPCP and vortioxetine was also associated with reduced affinity of the GABA_A_ receptor orthosteric site-selective ligand [^3^H]muscimol. Finally, subchronic PCP induced a reduction in GABA_B_ receptor expression that was insensitive to subchronic treatment with vortioxetine. However, we find that the relevance of these changes in GABA receptor biology for cognitive function is unclear.

### Vortioxetine Attenuates SubPCP-Induced Impairments in the Attentional Set-Shifting Task

Consistent with literature reports, subPCP treatment induced a reliable impairment in AST performance that was selective for the ED discrimination, and induced less reliable EDR discrimination impairments (Rodefer et al., 2005, 2008; Goetghebeur and Dias, 2009; Redrobe et al., 2012). Also consistent with literature findings, acute modafinil administration reliably reversed subPCP-induced impairments in the ED discrimination (Redrobe et al., 2012).

Acute vortioxetine administration (1, 3, and 10 mg/kg, 1 h s.c.), which encompasses the majority of the clinically relevant dose range of 50–90% SERT occupancy (Stenkrona et al., 2013; Leiser et al., 2014) significantly attenuated the subPCP-induced deficit in the ED but not the EDR discrimination. Although, it should be noted that the 10 mg/kg dose was able to attenuate subPCP-induced impairments in the EDR in one of the two experiments in which it was used. Subchronic vortioxetine administration at 0.22 g vortioxetine/kg of food weight (p.o. *ad libitum*) and 1.8 g vortioxetine/kg of food weight, which covers the entire clinically relevant dose range (Wallace et al., 2014; Waller et al., 2017), blocked subPCP-induced impairments in both the ED and EDR discrimination.

The fact that low vortioxetine doses (acute 1 mg/kg, sub-chronic 0.22 g/kg food) attenuated the effects of subPCP on AST performance may provide a hint at the mechanisms underlying its beneficial effects in this model. At the 1 mg/kg acute dose, vortioxetine exhibits full occupancy at the 5-HT_3_ receptor, where it acts as an antagonist, and 60–65% occupancy at the SERT (Leiser et al., 2014). Similarly, the 0.22 g/kg subchronic dose exhibit full occupancy at 5-HT_3_ receptors and approximately 50% occupancy at the SERT (SERT occupancy: Waller et al., 2017; 5-HT_3_ receptor occupancy estimated based on known SERT:5-HT_3_ receptor occupancy relationships; Leiser et al., 2014). In each case, occupancy at vortioxetine’s other receptor targets is negligible. To assess the relevance of these two mechanisms a separate experiment was undertaken using the selective 5-HT_3_ receptor antagonist ondansetron (1.6 mg/kg 1 h s.c., estimated 5-HT_3_ receptor occupancy 60–65%; du Jardin et al., 2014), and the selective SERT inhibitor escitalopram (0.5 mg/kg 1 h s.c., estimated SERT occupancy 90%; Jensen et al., 2014) on subPCP-induced AST impairments. Acute administration of either ondansetron or escitalopram were independently capable of reversing subPCP-induced impairments in this task.

### 5-HT_3_ Receptor Antagonism: A Possible Role for Suppressing Non-parvalbumin Expressing GABAergic Interneurons in Attenuating SubPCP-Induced Cognitive Deficits

Ondansetron’s ability to reverse subPCP-induced impairments is particularly interesting. 5-HT_3_ receptors in the frontal cortex are thought to be exclusively expressed on a subset of non-PVIR GABAergic interneurons (Puig et al., 2004), where they act as a source of serotonin-mediated excitatory drive. 5-HT_3_ receptor antagonism is thought to suppress the GABAergic cell firing and drive down inhibitory postsynaptic currents (IPSCs) from pyramidal neurons (Dale et al., 2014), and thereby increase cortical pyramidal neuron firing (Riga et al., 2016). Similar effects have been observed with acute or subchronic vortioxetine treatment (Riga et al., 2016, 2017), but not escitalopram (Riga et al., 2017). Taken together with the effects of ondansetron in subPCP-treated animals in the AST task, these data open for the possibility that suppressing GABAergic neurotransmission stemming from non-PVIR sources mediates reversal of subPCP-induced cognitive impairments. This concept is further supported by data showing that negative allosteric modulation of α5 subunit-containing GABA_A_ receptors using RO4938581 significantly attenuated subPCP-induced impairments in the ED and EDR discriminations of the AST, using the same subPCP model featured in the current work (Redrobe et al., 2012).

The idea that suppressing GABA neurotransmission improves AST performance in subPCP-treated animals does not require that subPCP-induced impairments are caused by increased GABAergic neurotransmission. However, recent data suggest that subPCP induces complex changes in GABAergic neurotransmission, including increases and decreases from separate neural populations. Nomura et al. (2016) found that subPCP treatment in mice led to a significant increase in the threshold for inducing long term potentiation (LTP; an electrophysiological phenomenon related to plasticity that is commonly associated with cognition) in the hippocampal CA1 region and significant increase in IPSCs recorded from CA1 pyramidal neurons, suggesting that at least some portion GABAergic inhibition is increased after subPCP treatment. Other research groups have also observed subPCP-induced LTP impairments in the CA1 and lateral amygdala using lower subPCP doses (Pollard et al., 2012). Interestingly, at least one research group has observed that subPCP induces a reduction in glucose metabolism in the medial prefrontal cortex in rats (Cochran et al., 2003). Given the connection between glutamate neurotransmission and glucose metabolism in the brain (e.g., Patel et al., 2005), reductions in glucose metabolism such as this could point to a reduction in principal cell activity after subPCP treatment. These data may also indirectly implicate a net bias toward inhibition in the balance of excitatory and inhibitory neurotransmission in this model. If these observations can be replicated, they may support a role for increased GABAergic neurotransmission from some cellular populations, although more study is required to appropriately address this concept. However, these data conflict with observations suggesting reduced GABAergic neurotransmission in the subPCP model. Suppressed expression of the calcium binding protein PV is a common observation in subPCP-treated animals, i.e., reduced number, density or intensity of staining of PV-IR cells (Schroeder et al., 2000; Cochran et al., 2003; Jenkins et al., 2010; Pollard et al., 2012; Redrobe et al., 2012). But the apparently conflicting data sets are not necessarily mutually exclusive. PV-IR and non-PV-IR cells are separate neural populations with different roles in information processing, and thus it is possible that subPCP treatment regulates each of these populations in complex and opposing ways, including suppressed GABAergic neurotransmission from PV-IR fast spiking cells directed at the soma and axon initial segment of principal cells, and perhaps increased GABAergic neurotransmission directed toward principal cell dendritic arbors. However, this concept is speculative and should be viewed with caution until more supporting data can be produced.

### A Role for SERT Inhibition in Regulating SubPCP-Induced AST Impairments?

As noted above, the vortioxetine doses used involved SERT occupancies in the range of 50–90%. In order to test the relevance of SERT inhibition for this task, we studied escitalopram 0.5 mg/kg (ED_80%occupancy_ = 0.3 mg/kg 1 h s.c.; Jensen et al., 2014) in the subPCP AST. Escitalopram significantly reversed subPCP-induced impairments.

To our knowledge, this is the first observation that a clinically relevant dose of a selective SERT inhibitor can ameliorate subPCP-induced AST deficits. Previous investigations have shown that chronic treatment with citalopram or escitalopram attenuates impairments in the AST ED discrimination induced by chronic unpredictable stress (Bondi et al., 2008), suggesting that escitalopram’s pharmacological properties can be relevant for this aspect of cognition under some biological conditions. However, we are not aware of any evidence suggesting the biological effects of chronic stress are similar to those induced by subPCP treatment.

We only identified one other study of the effects of SERT inhibition on subPCP induced cognitive impairments. Hashimoto et al. (2007) observed that single supra-pharmacological doses of fluvoxamine and paroxetine (20 and 10 mg/kg; respectively; SERT occupancy ED_80_ ≈ 1.8 and 0.2 mg/kg, respectively; Pehrson et al., 2015) improved subtle subPCP-induced impairments in OR performance. Repeated administration of fluvoxamine but not paroxetine reversed deficits in OR performance. Hashimoto et al. (2007) attributed this effect of fluvoxamine to its agonist actions at the sigma_1_ receptor, since the sigma_1_ receptor antagonist NE-100 blocked this effect. We regard it as unlikely that a sigma_1_ receptor-mediated mechanism is relevant for escitalopram’s effects on subPCP-induced deficits at the 0.5 mg/kg dose in the AST. Escitalopram’s Ki -values for the SERT and sigma 1 receptors are reported to approximately 1 nM and 100 nM, respectively (Sanchez et al., 2003). Based on these data and previous work showing a SERT occupancy ED_50_ ≈ 0.07 mg/kg for escitalopram (Jensen et al., 2014; Pehrson et al., 2015), we roughly estimate escitalopram’s ED_50_ at the sigma_1_ receptor in the range of 6–8 mg/kg, or more than 10-fold above the dose used here. Thus, we expect escitalopram’s occupancy of the sigma_1_ receptor to be negligible and consider SERT inhibition the most likely mechanism through which escitalopram is acting in this condition. Whether escitalopram’s effects in this model are due to a downstream effect on GABAergic and/or glutamatergic neurotransmission cannot be discerned based on the data presented here. However, previous research demonstrates that acute treatment with SERT inhibitors, such as escitalopram (Riga et al., 2016) and fluoxetine (Gronier and Rasmussen, 2003) does not significantly alter frontal cortex pyramidal neuron firing, making it unlikely that escitalopram’s effects in this cognitive task are mediated via changes in glutamate neurotransmission. These data, along with the consistent improvements induced by modafinil, suggest that multiple mechanistic avenues for improving subPCP-induced AST impairments exist.

### Vortioxetine Effects on Object Recognition and Preference Tasks

Object recognition and object placement tasks were used as secondary endpoints designed to examine whether vortioxetine would ameliorate cognitive tasks disrupted by subPCP treatment but primarily mediated by brain regions other than the frontal cortex. Consistent with previous literature, these experiments found that subPCP treatment significantly impaired performance in the OR task in both rats and mice, and in the object placement task in mice (Horiguchi et al., 2011; Redrobe et al., 2012). Acute vortioxetine treatment attenuated subPCP-induced OR impairments in rats and subchronic vortioxetine attenuated subPCP-induced OR and placement impairments in mice. In our hands, vortioxetine has demonstrated a relatively consistent ability to improve performance in the OR task across different impairment models, including 5-HT-depletion (du Jardin et al., 2014; Jensen et al., 2014), and acute scopolamine administration (Pehrson et al., 2016a). We have also observed that vortioxetine counteracts poor OR performance induced by long retention intervals in normal rats (Mork et al., 2013), however, this effect was modest, and as demonstrated in the mouse OR and placement experiments presented here, vortioxetine does not have a consistent ability to improve performance over control animals.

These experiments were not designed to investigate the effects of specific receptor mechanisms in treating subPCP-induced OR or placement impairments, and thus we have a limited ability to discuss such issues here. However, from previous work on vortioxetine’s receptor occupancy profile, we can make some general assertions about the targets vortioxetine likely engage at the doses used here. The acute vortioxetine dose used in the rat OR task (10 mg/kg 1 h s.c.) engenders full occupancy (≥80%) at 5-HT_3_ receptors, SERT, and 5-HT_1B_ receptors, and lower but potentially active occupancy levels at 5-HT_1A_ and 5-HT_7_ receptors (≈40%; (Leiser et al., 2014). The subchronic vortioxetine dose used in the mouse OR and object placement tasks engenders full occupancy at 5-HT_3_ receptors and SERT, and approximately 50% occupancy at 5-HT_1B_ receptors (Wallace et al., 2014; Waller et al., 2017). Occupancy at 5-HT_1A_ and 5-HT_7_ receptors is expected to be negligible at this dose.

Thus, these data confirm that vortioxetine is capable of attenuating subPCP-induced impairments in more than one cognitive domain, and this action appears consistent across species. However, without more data using multiple vortioxetine doses and selective pharmacological ligands, we cannot comment on which receptor mechanisms are relevant for vortioxetine’s effects.

### Autoradiographic Data: SubPCP-Induced Increases in GABA_A_ Receptor Expression or Binding Were Not Replicated

Several published studies suggest that subchronic NMDA receptor antagonism changes GABA_A_ receptor function. For example, i.c.v. infusion of MK-801 (1 pmol/10 μL/h) for 7 days elicited a significant increase in GABA_A_ receptor binding; assessed using the orthosteric agonist [^3^H]-muscimol, and the benzodiazepine allosteric modulator [^3^H]-flunitrazepam in the cortex and CA3 sub-region of the hippocampus (Kim et al., 2000). These data could be interpreted either as increases in receptor expression or affinity. Similarly, Beninger et al. (2010) observed a significant increase in the expression of GABA_A_ receptors (Bmax using [^3^H]muscimol as ligand) in the frontal cortex and hippocampus, along with a reduction in the affinity of [^3^H]muscimol for GABA_A_ receptors. Despite the use of a similar subPCP dosing regimen, we did not replicate the observation of a subPCP-induced increase in cortical [^3^H]muscimol Bmax (**Table 3**) seen in Beninger et al. (2010). Thus, the consistency of subPCP-induced increases in GABA_A_ receptor expression is questionable. However, we observed a significant reduction in [^3^H]-muscimol affinity for GABA_A_ receptors in the subPCP/vortioxetine treated group, and a statistically significant (≈2.5-fold) increase in the affinity of the GABA_A_ receptor for GABA in the vehicle/vortioxetine group. Finally, we observed a significant reduction in the expression of GABA_B_ receptors in subPCP-treated animals that was not altered by vortioxetine treatment. The relevance of changes in GABA_B_ receptor expression to cognitive function is unclear, given that this vortioxetine dose reversed subPCP-induced impairments in the AST but did not affect reductions in frontal cortex GABA_B_ receptor expression.

Assuming that vortioxetine suppresses some aspects of GABAergic neurotransmission, then the increase in GABA_A_ receptor affinity for the endogenous agonist observed in the vehicle/vortioxetine group could be interpreted as a compensatory adaptation meant to attenuate vortioxetine’s actions on GABA neurotransmission. A similar argument could be made that the subPCP-induced reduction in GABA_B_ receptor expression is a homeostatic response to increased GABA_B_ receptor-mediated neurotransmission. However, given the inconsistencies in the effects of repeated NMDA receptor antagonism between this report and those in the published literature, we caution against using these ideas until these data can be replicated.

### Study Limitations and Future Studies

This study has several limitations that must be taken into account. First, vortioxetine exhibits an approximately 10-fold lesser affinity at rodent 5-HT_1A_ and 5-HT_7_ receptors by comparison to humans. Therefore, the effects of 5-HT_1A_ receptor agonism and 5-HT_7_ receptor antagonism have been underestimated in these studies. Thus, the degree to which these concepts or mechanisms translate to humans is unknown. More work is required to fully investigate which of vortioxetine’s mechanisms are relevant for attenuating subPCP-induced cognitive impairments, particularly in the OR and object placement tests. Moreover, there have been some suggestions that drugs acting on 5-HT_1A_ receptors are relevant for some types of cognition in schizophrenic patients (Sumiyoshi et al., 2013), and thus future studies should examine the role of 5-HT_1A_ receptor agonism in vortioxetine’s effects in this model. Finally, as these data are a first demonstration of vortioxetine’s effects in the subPCP model, an independent replication is necessary before the concepts we have advanced can be fully accepted.

## Conclusion

In conclusion, acute or subchronic treatment with the multimodal antidepressant vortioxetine significantly attenuates subPCP-induced impairments in animal models of executive function and memory. In addition, 5-HT_3_ receptor antagonism and SERT inhibition can independently mimic vortioxetine’s effects on executive function. Given the many reported aberrations observed in GABA and glutamate neurotransmission after subPCP treatment, and separate demonstrations that vortioxetine indirectly enhances glutamate neurotransmission via a 5-HT_3_ receptor-dependent suppression of GABAergic inhibitory drive, the data presented here further support the notion that vortioxetine’s downstream effects on GABA and glutamate neurotransmission are relevant for its positive effects on cognitive function. However, we do not regard this level of support as definitive; more replication and extension of these data is required to fully confirm this hypothesis. In addition, the observations that acute escitalopram and modafinil can improve AST performance in subPCP-treated animals suggest that multiple mechanisms can be used to attenuate subPCP-induced cognitive impairments.

## Ethics Statement

Experiments conducted in the United States of America were carried out in accordance with the recommendations of the Guide for the Care and Use of Laboratory Animals, and were approved by the Lundbeck Research USA, Inc. Institutional Animal Care and Use Committee prior to the start of the study. All other studies were carried out in accordance with Danish legislation regulating animal experiments, including Law and Order on Animal experiments Act No. 474 of 15/05/2014, Order No. 12 of 07/01/2016, and European Commission Directive 86/609/EEC. These studies were approved with a specific license from the Danish National Authority prior to the start of experiments.

## Author Contributions

AP and CP were involved in experimental design of the study, collection and analysis of data, and played a role in writing and editing the manuscript. KT was involved in the collection and analysis of the data and played a role in writing the manuscript. CS was involved in experimental design of the study, analysis of data, and played a role in writing and editing the manuscript.

## Conflict of Interest Statement

CP is an employee of H. Lundbeck A/S. AP, KT, and CS are former Lundbeck employees.
