# Supplementary material for: Vortioxetine Treatment Reverses Subchronic PCP Treatment-Induced Cognitive Impairments: A Potential Role for Serotonin Receptor-Mediated Regulation of GABA Neurotransmission
Source: Front Pharmacol. 2018 Mar 6;9:162. doi: 10.3389/fphar.2018.00162 (PMC5845537; doi:10.3389/fphar.2018.00162)
Supplement: Supplementary file 1 [file Data_Sheet_1.pdf]

Supplementary table 1. Total exploration time observed in rat novel object recognition experiments. Data are expressed as mean exploration time (in seconds) ± SEM. No significant treatment group differences were observed.

Group	Training Trial	Testing Trial
Veh/Veh	39.1 ± 4.2	40 ± 5.9
PCP/Veh	34.6 ± 2.8	33.8 ± 5
PCP/10Vor	34.1 ± 2.8	30 ± 3.2

Supplementary table 2. Total exploration times observed in mouse object recognition and placement experiments. Data are expressed as mean exploration time (in seconds) ± SEM. No significant treatment group differences were observed.

Group	Novel Object Recognition		Novel Object Placement	
	Training Trial	Testing Trial	Training Trial	Testing Trial
Veh/Veh	23.4 ± 3.7	18.8 ± 2.6	56.3 ± 4.8	34.8 ± 2.7
PCP/Veh	26.9 ± 3.2	12.1 ± 2.0	62.7 ± 5.4	33.4 ± 5.2
Veh/Vor	20.9 ± 2.9	11.7 ± 2.7	63.8 ± 3.7	40.5 ± 3.6
PCP/Vor	23.4 ± 2.8	11.8 ± 1.8	63.9 ± 4.8	36.1 ± 4.3
